# Methylation age acceleration does not predict mortality in schizophrenia

**DOI:** 10.1038/s41398-019-0489-3

**Published:** 2019-06-04

**Authors:** Kaarina Kowalec, Eilis Hannon, Georgina Mansell, Joe Burrage, Anil P. S. Ori, Roel A. Ophoff, Jonathan Mill, Patrick F. Sullivan

**Affiliations:** 1 Department of Medical Epidemiology and Biostatistics, Karolinska Institutet, Stockholm, Sweden; 2 College of Pharmacy, University of Manitoba, Winnipeg, Canada; 3 University of Exeter Medical School, Exeter, UK; 4 UCLA Center for Neurobehavioral Genetics, University of California, Los Angeles, CA USA; 5 Departments of Genetics and Psychiatry, University of North Carolina at Chapel Hill, Chapel Hill, NC USA

**Keywords:** Epigenetics and behaviour, Predictive markers, Epigenetics and behaviour, Predictive markers

## Abstract

Schizophrenia (SCZ) is associated with high mortality. DNA methylation levels vary over the life course, and pre-selected combinations of methylation array probes can be used to estimate “methylation age” (mAge). mAge correlates highly with chronological age but when it differs, termed mAge acceleration, it has been previously associated with all-cause mortality. We tested the association between mAge acceleration and mortality in SCZ and controls. We selected 190 SCZ cases and 190 controls from the Sweden Schizophrenia Study. Cases were identified from the Swedish Hospital Discharge Register with ≥5 specialist treatment contacts and ≥5 antipsychotic prescriptions. Controls had no psychotic disorder or antipsychotics. Subjects were selected if they had died or survived during follow-up (2:1 oversampling). Extracted DNA was assayed on the Illumina MethylationEPIC array. mAge was regressed on age at sampling to obtain mAge acceleration. Using Cox proportional hazards regression, the association between mAge acceleration and mortality was tested. After quality control, the following were available: *n* = 126 SCZ died, 63 SCZ alive, 127 controls died, 62 controls alive. In the primary analyses, we did not find a significant association between mAge acceleration and SCZ mortality (adjusted *p* > 0.005). Sensitivity analyses excluding SCZ cases with pre-existing cancer demonstrated a significant association between the Hannum mAge acceleration and mortality (hazard ratio = 1.13, 95% confidence interval = 1.04–1.22, *p* = 0.005). Per our pre-specified criteria, we did not confirm our primary hypothesis that mAge acceleration would predict subsequent mortality in people with SCZ, but we cannot rule out smaller effects or effects in patient subsets.

## Introduction

Schizophrenia (SCZ) is associated with significantly higher mortality and 12–15 years decreased life expectancy compared to healthy individuals^1^. The causes of increased mortality in SCZ may include internal (i.e., genetic risk) and external factors, such as adverse health behaviors (i.e., smoking) or the under-diagnosis and under-treatment of common causes of mortality, including cancer and ischemic heart disease^1^. Another possibility for increased mortality relates to the hypothesis of accelerated aging, that is, factors associated with the disorder accelerates changes in biological status. This has been investigated previously in major depressive disorder, using a DNA methylation-based method^2^. DNA methylation in nucleated blood cells varies over the course of life^3–5^, with levels across multiple sites used to derive an estimate of “DNA methylation age” (mAge), which correlates highly with chronological age^6–8^. The deviation between mAge and chronological age (i.e., accelerated mAge) has been identified as a potential risk factor for age-related diseases and all-cause mortality in non-psychiatric studies^6–10^.

Individuals with major depressive disorder have exhibited mAge acceleration in whole blood and post-mortem brain tissues suggesting accelerated aging^2^. Studies investigating the accelerated aging hypothesis in post-traumatic stress disorder and mAge have yielded mixed results. A meta-analysis of 2,186 individuals found no association between advanced mAge and lifetime trauma exposure or post-traumatic stress disorder^11^ and accelerated mAge was marginally associated with childhood trauma (*p* = 0.028) and lifetime post-traumatic stress disorder severity (*p* = 0.016)^11^. In SCZ, accelerated aging according to mAge has been investigated, however findings using Horvath mAge estimates in either brain tissue^12–15^ or blood samples^16^ have been insignificant. These previous studies of mAge acceleration and SCZ did not focus specifically on mortality and were also limited to the use of a single mAge estimator (Horvath).

mAge can be estimated from Illumina methylation arrays using different algorithms, principally the Hannum^6^, Horvath^8^, and Levine^9^ estimators. The Hannum estimator was developed using 71 CpG sites from adult whole blood samples^6^. This contrasts with the Horvath estimator, which was developed using 353 CpG sites from multiple tissues from children and adults^8^. The most recent is the Levine mAge estimator, which incorporates clinical measures to capture phenotypic differences associated with health span and lifespan along with 513 CpG probes in adults^9^. The Horvath mAge calculator has been used frequently in scientific studies due to ease of calculation and multi-tissue applicability; however, many studies, including a recent investigation into the association between mAge acceleration and mortality, used both Hannum and Horvath methods and found high agreement between the estimators^7^.

To the best of our knowledge, a specific investigation into whether accelerated mAge is associated with subsequent mortality in SCZ has not been done and may help inform or predict risk of mortality in individuals with SCZ. Here, using a sample from Sweden^17^, we tested the association between mAge acceleration and mortality in individuals with SCZ and controls. Critically, this was an older cohort as the mean age at sampling was in the mid-50 s and followed for a median of 6 years. Based on the literature^7,10,18^, we hypothesized that accelerated mAge would predict subsequent death in middle-aged individuals with SCZ, when compared to SCZ who were alive and compared to controls.

## Methods and materials

### Study design and study population

Our study is a case-control sample of participants in the Sweden Schizophrenia Study (S3)^17^. S3 is a population-based cohort of individuals born in Sweden including 4,936 SCZ cases and 6,321 healthy controls recruited between 2004–2010. SCZ cases were identified from the Swedish Hospital Discharge Register^19,20^ with ≥2 hospitalizations with a discharge diagnosis of SCZ or schizoaffective disorder (SAD)^21^. This operational definition of SCZ was validated in clinical, epidemiological, genetic epidemiological, and genetic studies^17^. More generally, the Hospital Discharge Register has high agreement with medical^19,20^ and psychiatric diagnoses^22^. Controls were also selected through Swedish Registers and were group-matched by age, sex and county of residence and had no lifetime diagnoses of SCZ, SAD, or bipolar disorder or antipsychotic prescriptions. In S3, a significantly higher proportion of those with SCZ died (*n* = 766, 15.5%) during follow up compared to controls who died (*n* = 197, 3.1%, *p* = 2 × 10^−120^).

We studied four groups: (A) SCZ who died at follow up, (B) SCZ still alive at follow up, (C) controls who died at follow up, and (D) controls still alive at follow up (Table 1). Possessing both alive and dead cases and controls is ideal as it will assist with investigating a potential ‘dose-response’ relationship of mAge acceleration and death^18^. We obtained the data for creating these groups from Swedish national registers. Redeemed antipsychotic drug prescriptions (Anatomical Therapeutic Chemical [ATC] Classification codes beginning only with N05A) were collected from the Sweden National Prescribed Drug Register^23^. Mortality was determined using the Swedish Cause of Death Register, which contains official causes of death using International Classification of Diseases (ICD)-10 codes (all deaths occurred after 1997)^24^. We over-sampled those who died in a 2:1 ratio as we were particularly interested in the association between mortality and mAge acceleration. Subjects were selected if they were between 45–65 years old at the time of sampling (there were insufficient numbers of controls who died, so we increased the upper age boundary for this group to 85 years). We excluded subjects, prior to selecting the samples for methylation arrays, who died by external, un-natural causes of death (e.g., accident or violence) as these causes of death may have been more stochastic and less connected to mAge acceleration. We reasoned that any advantages of including these subjects (i.e., these deaths might be related to SCZ indirectly leading to dangerous behavior and aggressiveness) were outweighed by the greater probability that un-natural deaths were unrelated to mAge acceleration.

**Table 1:** Subject groups for analysis

	SCZ-died	SCZ-alive	Control-died	Control-alive
Criteria	SCZ with ≥5 specialist treatment contacts for SCZ or SAD & ≥5 antipsychotic prescriptions		No lifetime SCZ, SAD, or bipolar disorder or antipsychotic prescriptions	
*N*	127	63	127	63
Age at sampling	45–65 years	45–65 years	45–85 years	45–65 years
Follow up	Died ≥1 year after sampling	Alive ≥3 years after sampling	Died ≥1 year after sampling	Alive ≥3 years after sampling
Exclusion	Removed deaths by suicide, accident, or violence	None	Removed deaths by suicide, accident, or violence	None

*SCZ* schizophrenia, *SAD* schizoaffective disorder

All subjects were 18 years of age or older and provided written informed consent. Ethical permission was obtained from the Karolinska Institutet Ethical Review Committee in Stockholm, Sweden.

### mAge estimation

Blood samples were drawn at enrolment. 500 ng of DNA from each sample was treated with sodium bisulfite, using the EZ-96 DNA methylation-Gold kit (Zymo Research, CA, USA). DNA methylation was quantified using the Illumina Infinium HumanMethylationEPIC BeadChip (866,562 methylation sites across the genome) (Illumina Inc, CA, USA) and run on an Illumina iScan System (Illumina, CA, USA) using the manufacturers’ standard protocol^25^. In addition, a fully methylated control (CpG Methylated HeLa Genomic DNA; New England BioLabs, MA, USA) was included in a random position on each plate to facilitate sample tracking, resolve experimental inconsistencies, and confirm data quality. To reduce chip effects, we block randomized samples by group, age at sampling, and sex in groups of eight to each methylation chip using the R package *OSAT*^26^. The output of this array is the ratio of methylated:unmethylated alleles to quantify a β-value ranging from 0 (completely unmethylated) to 1 (completely methylated). Subjects were removed if methylated and unmethylated signal intensities < 2000, bisulfite conversion < 80%, sex mismatch, and/or if >1% of probes had detection *p*-value < 0.05. Methylation probes were removed if >1% of samples have detection *p*-value < 0.05 or if >5% of samples had a bead count < 3. The methylation data were then quantile-normalized using the dasen function from the *wateRmelon* R package^27^. As methylation data can be subject to measurement variation, we also normalized the methylation data using the *ENmix* R package^28^, which employs a mixture of exponential and truncated normal distributions to model background noise. Two samples whose predicted sex did not match their reported sex were excluded during quality control (1 SCZ died, 1 control alive), leaving a total of 378 samples and 809,996 methylation probes for analysis.

DNA methylation is robustly associated with tobacco smoking^29^. A per-individual smoking score (continuous measure) was generated based on DNA methylation sites known to be associated with smoking^30^. Using this method, current smokers of European ancestry have been identified with 100% sensitivity and 97% specificity^30^. To account for methylation differences between cell types, we estimated the cell-type composition of the blood samples using the function estimateCellCounts (*minfi* R package)^31^.

### Statistical analyses

mAge was computed using three different algorithms: Hannum^6^ based on 71 methylation probes, Horvath^8^ using 353 probes, and Levine^9^ using 513 probes. The residuals resulting from a linear regression of mAge on age at sampling were used as the measure of mAge acceleration for each mAge estimator. A positive value indicates that the blood sample of the individual is older than that of their chronological age. Horvath mAge was calculated using the DNAmAge software (http://labs.genetics.ucla.edu/horvath/dnamage), which includes the use of an additional normalization step as part of its procedures. Hannum and Levine mAge were estimated using the coefficients listed by Hannum et al.^6^ and Levine et al^9^.

Cox proportional hazards regression analyses (R packages: *survival*, *My*.*stepwise*) were performed to compare the mAge acceleration (separately for the three mAge estimators) between: SCZ-died vs. SCZ alive; SCZ-died vs. controls-died; and SCZ-died vs. controls alive. Hazard ratios and corresponding 95% confidence intervals were reported. Hazard ratios were adjusted for known mortality risk factors (sex, age, smoking score, and white blood cell composition) and for covariates known to affect methylation (smoking score, array batch, and white blood cell composition). We examined the Schoenfeld residuals to test the proportional hazards assumption and the hazards for mortality were proportional over time (*p* = 0.65). As a complementary analysis, we computed the least square means (*emmeans* R package) in two linear models: mAge (i.e., predicted mAge) and mAge acceleration (i.e., mAge residuals). These linear models included case/control status, died/alive, and their interaction term along with the following covariates: sex, methylation batch, white blood cell counts, and smoking score. The mAge least square mean was additionally adjusted for age at sampling.

We performed sensitivity analyses. First, to assess the influence of acute illness on mAge acceleration, we removed all deaths that occurred < 2 years of sampling. Second, we removed all individuals who were >65 years at the time of sampling from the control-died group, given the other subject groups were limited to ≤ 65 years. Third, we removed all individuals with any previous inpatient or outpatient contacts for a serious cancer (ICD codes for serious cancers listed in Supplementary Table S1), given the strong evidence for epigenetic changes in cancer^32^. Lastly, we removed all individuals with any previous contacts for a serious cancer and were >65 years at the time of sampling. For these sensitivity analyses, we performed a Cox proportional hazards regression analysis to compare findings with the results from the main analysis.

All analyses were performed using *R* (version 3.3.3) and R-Studio (version 1.0.143). We chose to adopt a more stringent *p*-value level (*p* ≤ 0.005) to indicate statistical significance^33^ and reduce the likelihood of a false positive finding.

## Results

Following quality control, the following groups were available for analysis of mAge acceleration and mortality: SCZ (*n*_died_ = 126, *n*_alive_ = 63) and controls (*n*_died_ = 127, *n*_alive_ = 62) (Table 2). The median ages at sampling were as expected across the four groups due to study design. As noted in the literature^1^ and due to our study design, SCZ cases had an earlier age at death, compared to controls (median = 62.3 y vs. 71.2 y). Subjects who died had approximately four years of follow-up time, compared to 5–6 years of follow up for those alive. Individuals with SCZ had a similar number of inpatient and outpatient hospitalizations between those who died compared to alive, although those SCZ cases who were alive had a higher number of antipsychotic prescriptions. The causes of death between SCZ and controls were compared (Supplementary Table S2), and as expected majority of deaths in both groups occurred from cardiovascular disease and cancer^1^. SCZ subjects who died had a higher median smoking score than the three other subject groups (Table 2), in addition to a higher proportion of SCZ died from respiratory diseases than controls. Therefore, smoking score was an important covariate to consider in the mAge acceleration analyses.

**Table 2:** Descriptive statistics for the four groups for analysis

	SCZ-died	SCZ-alive	Control-died	Control-alive
*N*	126	63	127	62
Males, *N* (%)	76 (60.3)	38 (60.3)	71 (55.9)	40 (63.5)
Age at sampling, years	58 (53, 61)	56 (51, 60)	68 (61, 74)	57.5 (51, 61)
Age at death, years	62.3 (56.3, 65)	N/A	71.2 (65.4, 78.4)	N/A
Follow-up time^a^	3.95 (2.9, 5.5)	6(5, 6)	4.1 (2.8, 5.4)	5(5, 6)
Inpatient hospitalizations^b^	13.5 (7.3, 24)	13 (7, 25.5)	0	0
Outpatient hospitalizations^b^	9.5 (5, 16.8)	12(5, 19)	0	0
Antipsychotic prescriptions	140 (40.5, 247)	205 (90.5, 341)	0	0
Smoking score	6.2 (1.1, 9.6)	2.3 (−1.8, 7.1)	−0.74 (−3.5, 3.4)	−2.3 (−4.6, 0.2)
Hannum				
mAge	53.7 (49.8, 56.7)	51.2 (48.4, 56.1)	60.7 (55.3, 65.9)	51.7 (49.1, 56.7)
mAge acceleration	−0.17 (−2.5, 2.1)	−0.40 (−2.3, 1.1)	0.04 (−2.2, 2.2)	0.20 (−2.8, 2.5)
Horvath				
mAge	57.1 (52.6, 61.5)	55.2 (51.9, 58.6)	64.6 (58.5, 69.9)	57.2 (53.4, 60.8)
mAge acceleration	0.31 (−3.2, 2.9)	−0.27 (−2.9, 1.5)	−0.35 (−2.8, 2.5)	0.29 (−1.9, 2.7)
Levine				
mAge	50.6 (45.9, 55.4)	47.8 (43.7, 53.3)	58.9 (52.4, 64.9)	48.2 (43.2, 52.4)
mAge acceleration	0.72 (−2.6, 3.6)	−0.88 (−3.4, 2.5)	0.04 (−3.5, 3.5)	−1.5 (−4.5, 3.1)

For continuous variables, the median and interquartile range are reported; for categorical variables, the number of individuals and percent are reported. For the methylation age (mAge), the value is reported in years ^a^For those who died, time between sampling and death; for alive, this is time between sampling and last follow-up ^b^Hospitalizations with discharge diagnoses of SCZ or schizoaffective disorder. *N/A* not applicable

### Methylation age acceleration and mortality

**Fig. 1:**
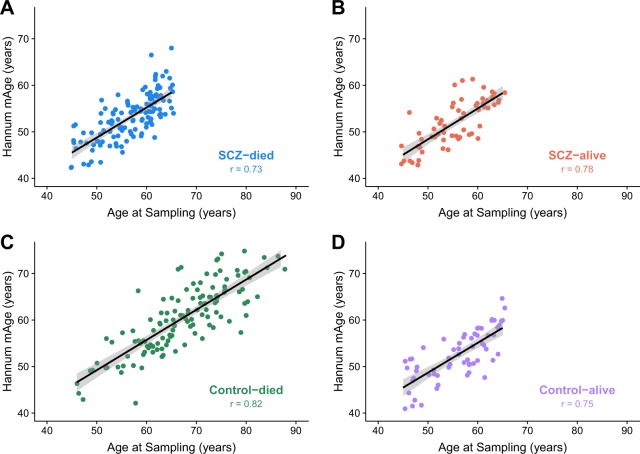
Plot of predicted Hannum methylation age (mAge) against age at sampling, by status: **a** SCZ-died, **b** SCZ-alive, **c** Control-died, **d** Control-alive. Correlation between age at sampling and Hannum mAge across all samples was 0.85. *r* = Pearson correlation coefficient, black line = linear model, gray region = 95% confidence intervals

There were high correlations between age at sampling and mAge for all three estimators (range for all samples by estimator = 0.79–0.85, Supplementary Figure S1; range by sample for Hannum estimator = 0.73–0.82, Fig. 1). Furthermore, there was significant overlap of the interquartile ranges for the mAge acceleration for all subject groups (Table 2). Choice of normalization method (quantile-normalization using the dasen function versus background correction using the *ENmix* package) for the methylation data did not differ and was highly correlated for each of the three mAge estimators (Horvath mAge: *r* = 0.99, Hannum mAge: *r* = 0.99, and Levine mAge: *r* = 0.98). We proceeded with the quantile-normalized methylation data for all subsequent analyses given its use in previous studies of mAge acceleration^11,34^.

Upon performing a Cox regression analyses for mortality in SCZ, we did not detect a significant association (all *p* > 0.005) between the three mAge acceleration estimators and mortality in SCZ in any of the following comparisons: SCZ-died vs. SCZ alive (Table 3), and SCZ-died vs. controls-died; SCZ-died vs. controls alive (Supplementary Table S3). The equivalent analysis in controls was also performed, with similar effect sizes seen in previous studies^10^, albeit non-significant due to the smaller sample sizes (Supplementary Table S3). Findings were similar for unadjusted and adjusted hazard ratios across all comparisons, and none reached statistical significance. Additionally, a stepwise bidirectional Cox regression model was used to examine the individual effects of white blood cell counts, age at sampling, sex, and smoking scores on the three mAge acceleration estimators and mortality in SCZ, which revealed that no factors remained independently significant (all *p* > 0.005, Supplementary Table S4).

**Table 3:** Cox regression analyses of mAge acceleration versus mortality in schizophrenia cases

mAge estimator	Unadjusted hazard ratio (95%CI)	Adjusted hazard ratio (95%CI)^a^
Hannum	1.04 (0.99–1.10), *p* = 0.11	1.07 (1.01–1.13), *p* = 0.02
Horvath	1.02 (0.98–1.07), *p* = 0.41	1.03 (0.98–1.08), *p* = 0.24
Levine	1.02 (0.98–1.05), *p* = 0.45	1.00 (0.96–1.05), *p* = 0.84

^a^Adjusted for white blood cell counts, age, sex, smoking score, and methylation batch. The sample for this analysis is *n* = 126 SCZ died vs. *n* = 63 SCZ alive

The least square means from an adjusted linear model of Hannum mAge in all individuals (*N* = 378) revealed no significant effects of case-control status (*p* = 0.284) or mortality (*p* = 0.925, Supplementary Table S5). Similar findings were observed for a linear model using mAge acceleration (i.e., residuals), removing the interaction term from both linear models, and repeating these analyses using the Horvath and Levine mAge estimators (all *p* ≥ 0.1). We tested the association between mAge acceleration and known risk factors for mortality using linear regression (Supplementary Table S6) and there were no factors significantly associated with mAge acceleration in SCZ.

### Sensitivity analyses

We removed 37 individuals who had died within the first two years of sampling (*n*_scz_ = 20; *n*_controls_ = 17) and repeated the Cox regression analyses, with no change to the pattern of results; no mAge acceleration estimator was significantly associated with mortality in SCZ (all *p* > 0.005, Supplementary Table S7). As a second sensitivity analyses, we removed 73 individuals who were >65 years at the time of sampling from the controls-died group (Supplementary Table S8). A significant result was identified in the unadjusted analyses for the Hannum estimator (hazard ratio = 1.06, 95% confidence interval = 1.02–1.11, *p* = 0.004), however, there was less certainty regarding this effect, upon adjusting for confounders (*p* = 0.009). The remaining findings from this sensitivity analyses were again in line with our primary findings, in addition to complementary analyses performed in controls only (*p* ≥ 0.2). We then removed 130 individuals (SCZ-died: *n* = 43, SCZ-alive: *n* = 7, control-died: *n* = 74, control-alive: *n* = 6) with any treatment contacts for a serious cancer that may affect mAge acceleration (Table 4). These sensitivity analyses revealed a significant finding for the Hannum mAge acceleration and mortality in SCZ (hazard ratio = 1.13, 95% confidence interval = 1.04–1.22, *p* = 0.005), although the 95% confidence intervals were broader in this smaller cohort, compared to the primary analyses. Finally, a sensitivity analyses was performed whereby we removed 147 individuals (SCZ-died: *n* = 43, control-died: *n* = 104) who were >65 years at the time of sampling and had any treatment contact for a serious cancer. The results for the mAge acceleration and SCZ and mortality were as previous (*p*-values adjusted for white blood cell counts, age, sex, smoking score, and methylation batch: Hannum [*p* = 0.016], Horvath [*p* = 0.441], Levine [*p* = 0.754]).

**Table 4:** Cox regression analyses of mAge acceleration versus mortality in schizophrenia and controls after excluding individuals with pre-existing cancer

	SCZ-died (*n* = 83) vs. SCZ-alive (*n* = 56)		SCZ-died (*n* = 83) vs. Controls-alive (*n* = 56)		SCZ-died (*n* = 83) vs. Controls-died (*n* = 53)	
	Unadjusted	Adjusted^a^	Unadjusted	Adjusted^a^	Unadjusted	Adjusted^a^
Hannum	1.06 (0.98–1.14); *p* = 0.09	1.13 (1.04–1.22); *p* = 0.005	1.04 (0.97–1.10); *p* = 0.27	1.10 (1.01–1.19); *p* = 0.02	1.03 (0.99–1.08); *p* = 0.18	1.05 (0.98–1.11); *p* = 0.12
Horvath	1.04 (0.97–1.10); *p* = 0.28	1.06 (0.99–1.13); *p* = 0.08	1.00 (0.95–1.07); *p* = 0.89	1.06 (0.99–1.14); *p* = 0.08	1.00 (0.96–1.04); *p* = 0.96	1.01 (0.97–1.06); *p* = 0.55
Levine	1.01 (0.97–1.07); *p* = 0.44	1.01 (0.96–1.07); *p* = 0.63	1.03 (0.98–1.08); *p* = 0.25	0.99 (0.94–1.05); *p* = 0.76	0.99 (0.96–1.04); *p* = 0.91	0.98 (0.94–1.03); *p* = 0.56

Values are Hazard Ratios (95% Confidence Intervals) and *p*-values

^a^Adjusted for white blood cell counts, age, sex, smoking score, and methylation batch

### Power calculation

Given our null results, it is important to determine the statistical power to detect a difference. For the key analyses of SCZ died vs. SCZ alive, we performed a power calculation using the R package *powerSurvEpi*. We had >80% power to detect the expected effect size for mAge acceleration and mortality in SCZ (*p* = 0.005, 189 subjects, proportion died = 0.66, expected hazard ratio = 1.17^18^). The expected hazard ratio is derived from a previous study reporting mAge acceleration was associated with mortality in cancer (hazard ratio = 1.17, 95%CI = 1.07–1.28)^18^. We hypothesized that this effect size was more appropriate than the smaller effects reported by the all-cause mortality studies of non-diseased individuals (e.g., Chen et al.^10^).

## Discussion

We conducted a well-powered case-control study within a Swedish study of SCZ and controls^17^, finding the evidence was too weak to support our pre-specified primary hypothesis that mAge acceleration is associated with mortality in SCZ. mAge acceleration has been previously associated with all-cause mortality, including in a large meta-analysis of 13,089 individuals (2734 deaths; *p* ≤ 8.2 × 10^−9^)^10^, with similar findings in smaller cohorts^5,7,35^. However, these previous studies used population-based cohorts of individuals with no specific disease and thus, it is possible that the underlying mechanism of mAge acceleration as a predictor of mortality differs in SCZ. Investigations of the association between mAge acceleration and mortality specifically in psychiatric disorders are currently lacking. Thus, to the best of our knowledge, this study represents the first of its kind in relation to mortality and SCZ.

Although the evidence was not strong enough to suggest that mAge acceleration is associated with mortality in SCZ, there remains the possibility of this association in tissues outside the blood, or smaller effects, particularly within patient subsets. For example, mAge acceleration has been noted in individuals with Down’s syndrome using brain and blood samples but not buccal epithelium^36^. However, SCZ studies not specifically investigating mortality that employed different brain tissues and blood found no evidence of mAge acceleration^12–16^. More specifically, these mAge acceleration studies in SCZ involved samples from the superior temporal gyrus (*N* = 22 SCZ, 22 controls)^12^ and frontal cortex (*N* = 199 SCZ, 241 controls)^13^ and found no association between mAge acceleration and SCZ. The lack of association was replicated in samples from dorsolateral prefrontal cortex (*N* = 195 SCZ, 232 controls; *p* = 0.60) and blood (*N* = 592 SCZ, 707 controls; *p* ≥ 0.1)^16^. There remains the possibility that since 25% of SCZ cases died in this study from cardiovascular disease, samples of heart tissue could point to a possible association between mAge acceleration and mortality in SCZ and presents an interesting avenue for future investigations.

We assessed the effects of multiple confounders on the association of mAge acceleration and mortality in SCZ. Owing to our study design, the age at sampling was similar for all subject groups, apart from the control-died group, who were older. However, there was evidence of residual age confounding by the change in the hazard ratio following the removal of older individuals (>65 years at sampling) from the control-died group during the sensitivity analyses. We also assessed the effects of acute illness on mAge acceleration by removing individuals who died within the first two years of sampling and the findings did not change. Removing individuals with a lifetime treatment contact for a serious cancer revealed a significant finding for the Hannum estimator, although this finding requires follow up in a larger cohort of individuals with SCZ and without cancer. This finding highlights the importance of having age-matched controls and excluding individuals with pre-existing cancer in investigating the association of mAge acceleration and mortality.

Our study had multiple strengths. We selected all subjects to be of a similar age at sampling to reduce confounding and we performed sensitivity analyses to exclude the possibility that individuals who were older at the time of sampling had influenced the results. The ability to ascertain the actual cause of death is a strength as it meant we were able to exclude subjects who died from external causes, as these would be less likely to be affected by mAge acceleration. Rather than employing logistic regression to assess the association between mAge acceleration and mortality in SCZ, our primary analyses involved a Cox proportional hazards regression model, which was able to assess simultaneously the effect of mAge acceleration on mortality and varying lengths of follow-up between subjects. Further, we employed multiple mAge estimators, including Hannum, which was developed for the same sample type used here (adult blood samples)^6^. A limitation of our study included an inability to assess the influence of additional confounders on the association of mAge acceleration and mortality, such as the use of valproic acid^37^, or patient characteristics, such as body mass index^38^.

In conclusion, in a sample of SCZ cases and controls with considerable follow-up, we found no evidence to suggest that mAge acceleration was associated with mortality. However, future studies in this area may consider investigating mAge acceleration in SCZ patient subsets without cancer or including longitudinal measurements of blood in SCZ to determine whether mAge acceleration changes over time^39^. Additionally, the advent of the single cell technologies may reveal a relationship between mAge acceleration and SCZ mortality by elucidating the individual cell type contributing to aging, tissue repair, or tissue rejuvenation in SCZ^40^.

## Supplementary information

Supplementary Information

Supplementary Figure Legend for Figure S1

Supplementary Figure S1

## Data Availability

Custom written R scripts used for statistical analyses can be provided upon request.
