# Supplementary material for: Methylation age acceleration does not predict mortality in schizophrenia
Source: Transl Psychiatry. 2019 Jun 4;9:157. doi: 10.1038/s41398-019-0489-3 (PMC6548770; doi:10.1038/s41398-019-0489-3)
Supplement: Supplementary file 1 — Supplementary Information [file 41398_2019_489_MOESM1_ESM.docx]

## **Supplemental Information**

***Supplementary Table S1:*** List of cancer diagnostic codes utilized for removing subjects with any serious lifetime cancer for the sensitivity analyses of mAge and mortality analyses.

| **Cause** | **ICD-code** |
| --- | --- |
| **Malignant neoplasms** | *ICD-9:* 153.4, 162.3, 174.0, 183  *ICD-10:* C03, C06, C09, C15-C22, C24, C25, C30, C32, C34, C37, C38, C43, C44, C49, C50, C53, C54-C57, C61, C64, C65, C67, C69, C71, C73, C74, C76-C80 |
| **Leukemia** | *ICD-9:* 204  *ICD-10:* C92, C95 |
| **Hodgkin's and non-Hodgkin's disease** | *ICD-10:* C81-C83, C85 |
| **Multiple myeloma and malignant plasma cell neoplasms** | *ICD-10:* C90 |
| **Carcinoma in situ of breast** | *ICD-10:* D059 |
| **Polycythemia vera** | *ICD-10:* D459 |
| **Myelodysplastic syndromes** | *ICD-10:* D469 |
| **Other neoplasms of uncertain or unknown behavior of lymphoid, hematopoietic, and related tissue** | *ICD-10:* D471, D474, D479, D485 |

***Supplementary Table S2:*** Causes of death in schizophrenia and controls from the Swedish cause of death register (1).

| Cause | ICD-10 codes | SCZ,  n=126, n (%) | Control,  n=127, n (%) |
| --- | --- | --- | --- |
| Cardiovascular disease | I00-I59, I70-I99 | 31 (24.6) | 34 (26.8) |
| Stroke | I60-I69 | 3 (2.4) | 5 (3.9) |
| Breast cancer | C50 | 2 (1.6) | 5 (3.9) |
| Prostate cancer | C61 | 0 | 6 (4.7) |
| Lung cancer | C34 | 11 (8.7) | 9 (7.1) |
| Other cancers | C00-C33, C35-C49, C51-C60, C62-C97 | 29 (23.0) | 42 (33.1) |
| Respiratory diseases | J00-J99 | 19 (15.1) | 7 (5.5) |
| Endocrine, nutritional, and metabolic diseases, and immune disorders | E00-E90  D80-D89 | 9 (7.1) | 6 (4.7) |
| Diseases of digestive system | K00-93 | 7 (5.5) | 1 (0.8) |
| Diseases of nervous system and sense organs | G00-99, H00-59, H60-95 | 2 (1.6) | 5 (3.9) |
| Infections and parasitic conditions | A00-B99 | 1 (0.8) | 2 (1.6) |
| Diseases of blood and blood-forming organs | D50-D77 | 0 | 1 (0.8) |
| Diseases of skin and subcutaneous tissue | L00-99 | 0 | 0 |
| Diseases of musculoskeletal system and connective tissue | M00-99 | 0 | 0 |
| Other | All other codes (excluding non-natural causes) | 12 (9.5) | 4 (3.1) |

*Supplementary Table S3:* Cox regression analyses of mAge acceleration versus mortality in schizophrenia and controls.

|  | **SCZ-died (n=126) vs.**  **Controls-alive (n=62)** | | **SCZ-died (n=126) vs.**  **Controls-died (n=127)** | | **Controls-died (n=127) vs. Controls-alive (n=62)** | |
| --- | --- | --- | --- | --- | --- | --- |
|  | **Unadjusted** | **Adjusted^1^** | **Unadjusted** | **Adjusted^1^** | **Unadjusted** | **Adjusted^1^** |
| **Hannum** | 1.03 (0.98-1.08);  *p*=0.25 | 1.05 (0.99-1.12);  *p*=0.09 | 1.04 (1.01-1.07);  *p*=0.02 | 1.03 (0.99-1.07);  *p*=0.14 | 1.02 (0.98-1.07);  *p*=0.32 | 0.98 (0.93-1.05); *p*=0.64 |
| **Horvath** | 0.99 (0.95-1.04);  *p*=0.79 | 1.03 (0.98-1.08);  *p*=0.26 | 1.00 (0.97-1.03);  *p*=0.97 | 0.99 (0.97-1.03);  *p*=0.88 | 0.98 (0.95-1.03);  *p*=0.57 | 1.00 (0.96-1.05); *p*=0.96 |
| **Levine** | 1.02 (0.98-1.06);  *p*=0.33 | 0.99 (0.95-1.04);  *p*=0.73 | 1.02 (0.99-1.05);  *p*=0.17 | 1.01 (0.98-1.04);  *p*=0.61 | 1.03 (0.99-1.06);  *p*=0.15 | 1.01 (0.96-1.05); *p*=0.81 |

Values are Hazard Ratios (95% Confidence Intervals); *p*-values. ^1^Adjusted for white blood cell counts, age, sex, smoking score, and methylation batch.

*Supplementary Table S4:* Stepwise bidirectional Cox regression analyses of mAge acceleration versus mortality in schizophrenia cases.

| **mAge estimator** | **Adjusted Hazard Ratio (95%CI)^1^** |
| --- | --- |
| **Hannum** | 1.06 (1.01-1.12), *p*=0.02 |
| **Horvath** | 1.04 (0.98-1.09), *p*=0.12 |
| **Levine** | 1.02 (0.98-1.06), *p*=0.41 |

^1^Covariates selected by the stepwise regression were white blood cell counts, age, and smoking score. The sample for this analysis is n = 126 SCZ died vs. n = 63 SCZ alive

*Supplementary Table S5:* Least square means analysis of mAge and mAge acceleration as predicted by case-control status or mortality in all study participants (N=378).

|  |  | **Interaction term included (case/control status x mortality)^1^** | | | | **Interaction term excluded^1^** | | | |
| --- | --- | --- | --- | --- | --- | --- | --- | --- | --- |
|  |  | **mAge** | | **mAge Acceleration** | | **mAge** | | **mAge Acceleration** | |
| **mAge estimator** | **Predictor** | **Beta (SE)** | **p-value** | **Beta (SE)** | **p-value** | **Beta (SE)** | **p-value** | **Beta (SE)** | **p-value** |
| **Hannum** | **Case-control status** | -0.63 (0.59) | 0.284 | -0.62 (0.59) | 0.297 | -0.31 (0.41) | 0.442 | 0.09 (0.39) | 0.817 |
|  | **Mortality** | -0.05 (0.56) | 0.925 | -0.81 (0.52) | 0.121 | 0.27 (0.39) | 0.486 | -0.21 (0.36) | 0.558 |
| **Horvath** | **Case-control status** | -0.47 (0.77) | 0.544 | -0.47 (0.77) | 0.544 | 0.06 (0.52) | 0.900 | 0.26 (0.51) | 0.609 |
|  | **Mortality** | -0.32 (0.74) | 0.669 | -0.62 (0.67) | 0.357 | 0.20 (0.50) | 0.683 | -0.03 (0.47) | 0.955 |
| **Levine** | **Case-control status** | -0.41 (0.89) | 0.645 | -0.39 (0.91) | 0.661 | -0.73 (0.61) | 0.234 | -0.17 (0.59) | 0.769 |
|  | **Mortality** | 0.65 (0.86) | 0.446 | -0.51 (0.79) | 0.524 | 0.35 (0.58) | 0.542 | -0.31 (0.55) | 0.576 |

^1^Adjusted for mortality, case/control status, white blood cell counts, age, sex, smoking score, and methylation batch. SE: Standard error.

*Supplementary Table S6:* Linear regression analyses of mAge acceleration versus known mortality risk factors.

|  | **SCZ-died (n=126) vs. SCZ-alive (n=63)** | | |
| --- | --- | --- | --- |
|  | **Beta** | **Standard error** | ***p*-value** |
| ***Hannum mAge acceleration*** |  |  |  |
| **Male sex** | 1.37 | 0.49 | 5.8x10^-3^ |
| **Age** | -0.01 | 0.04 | 0.76 |
| **Smoking score** | 0.04 | 0.04 | 0.31 |
|  | | | |
| ***Horvath mAge acceleration*** |  |  |  |
| **Male sex** | -0.49 | 0.58 | 0.40 |
| **Age** | 0.06 | 0.05 | 0.19 |
| **Smoking score** | 0.04 | 0.05 | 0.43 |
|  | | | |
| ***Levine mAge acceleration*** |  |  |  |
| **Male sex** | -0.85 | 0.76 | 0.26 |
| **Age** | 0.03 | 0.07 | 0.67 |
| **Smoking score** | 0.13 | 0.06 | 3.2x10^-2^ |

Values are unadjusted.

*Supplementary Table S7:* Cox regression analyses of mAge acceleration versus mortality in schizophrenia and controls after removing deaths that occurred within two years after sampling.

|  | **SCZ-died (n=106) vs.**  **SCZ-alive (n=63)** | | **SCZ-died (n=106) vs.**  **Controls-alive (n=62)** | | **SCZ-died (n=106) vs.**  **Controls-died (n=110)** | |
| --- | --- | --- | --- | --- | --- | --- |
|  | **Unadjusted** | **Adjusted^a^** | **Unadjusted** | **Adjusted^a^** | **Unadjusted** | **Adjusted^a^** |
| **Hannum** | 1.03  (0.97-1.09);  *p*=0.30 | 1.05  (0.98-1.13);  *p*=0.12 | 1.02  (0.96-1.07);  *p*=0.55 | 1.03  (0.97-1.10);  *p*=0.32 | 1.04  (1.00-1.07);  *p*=0.04 | 1.03  (0.99-1.08);  *p*=0.17 |
| **Horvath** | 1.03  (0.98-1.09);  *p*=0.18 | 1.04  (0.99-1.10);  *p*=0.12 | 1.01  (0.96-1.06);  *p*=0.78 | 1.04  (0.98-1.10);  *p*=0.14 | 1.00  (0.98-1.04);  *p*=0.81 | 1.00  (0.97-1.04);  *p*=0.96 |
| **Levine** | 1.01  (0.97-1.06);  *p*=0.51 | 1.00  (0.95-1.05);  *p*=0.90 | 1.01  (0.98-1.07);  *p*=0.44 | 0.98  (0.94-1.04);  *p*=0.56 | 1.02  (0.99-1.05);  *p*=0.26 | 1.01  (0.98-1.04);  *p*=0.65 |

Values are Hazard Ratios (95% Confidence Intervals) and *p*-values. ^a^Adjusted for white blood cell counts, age, sex, smoking score, and methylation batch.

*Supplementary Table S8:* Cox regression analyses of mAge acceleration versus mortality in schizophrenia and controls after removing individuals that were >65 years at the time of sampling.

|  | **SCZ-died (n=126) vs.**  **Controls-died (n=54)** | | **Controls-died (n=54) vs.**  **Controls-alive (n=62)** | |
| --- | --- | --- | --- | --- |
|  | **Unadjusted** | **Adjusted^1^** | **Unadjusted** | **Adjusted^1^** |
| **Hannum** | 1.06 (1.02-1.11), *p*=0.004 | 1.07 (1.02-1.13), *p*=0.009 | 1.02 (0.95-1.10), *p*=0.57 | 0.98 (0.88-1.09), *p*=0.72 |
| **Horvath** | 1.00 (0.97-1.04), *p*=0.94 | 1.00 (0.97-1.05), *p*=0.86 | 0.96 (0.90-1.04), *p*=0.34 | 0.98 (0.91-1.07), *p*=0.72 |
| **Levine** | 1.01 (0.98-1.05), *p*=0.35 | 1.01 (0.97-1.04), *p*=0.67 | 1.02 (0.98-1.08), *p*=0.29 | 1.01 (0.96-1.08), *p*=0.59 |

Values are Hazard Ratios (95% Confidence Intervals) and *p*-values. ^1^Adjusted for white blood cell counts, age, sex, smoking score, and methylation batch.

**References for Supplemental Information**

1. Brooke HL, Talbäck M, Hörnblad J, Johansson LA, Ludvigsson JF, Druid H, *et al.* (2017): The Swedish cause of death register. *Eur J Epidemiol*. 32: 765–773.
