# Supplementary material for: Methylation age acceleration does not predict mortality in schizophrenia
Source: Transl Psychiatry. 2019 Jun 4;9:157. doi: 10.1038/s41398-019-0489-3 (PMC6548770; doi:10.1038/s41398-019-0489-3)
Supplement: Supplementary file 2 — Supplementary Figure Legend for Figure S1 [file 41398_2019_489_MOESM2_ESM.docx]

***Supplementary Figure S1:*** Plot of (A) Horvath methylation age (mAge) against age at sampling, (B) Levine mAge against age at sampling, (C) Levine versus Hannum mAge, (D) Levine versus Horvath mAge and (E) Hannum versus Horvath mAge. r = Pearson correlation coefficient, black line = linear model, gray region = 95% confidence intervals.
