# Supplementary figures and images for: Methylation age acceleration does not predict mortality in schizophrenia
Source: Transl Psychiatry. 2019 Jun 4;9:157. doi: 10.1038/s41398-019-0489-3 (PMC6548770; doi:10.1038/s41398-019-0489-3)

Supplementary Figure 1

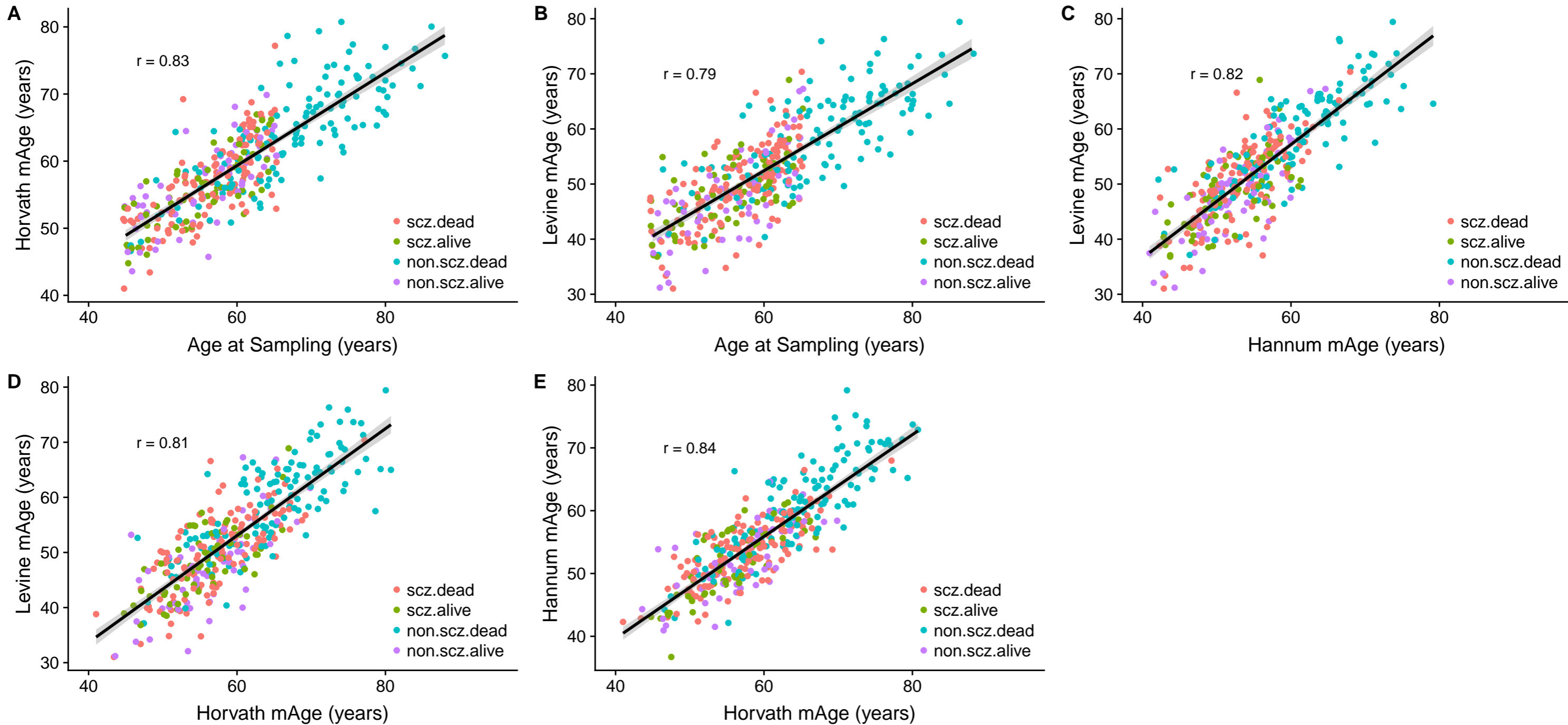

Supplement: Supplementary file 3 — Supplementary Figure S1 [file 41398_2019_489_MOESM3_ESM.pdf]
